# Integrated metabolomics, transcriptomic, and phytohormonal analyses to study the effects of water stress and foliar abscisic acid application in *Thymus* species using LC-MS/MS

**DOI:** 10.3389/fpls.2025.1557446

**Published:** 2025-03-11

**Authors:** Maryam Alipour, Maryam Haghighi, Mehdi Rahimmalek, Michael Reichelt, Laure Martinelli, Karin Groten, Axel Mithöfer

**Affiliations:** ^1^ Department of Plant Defense Physiology, Max Planck Institute for Chemical Ecology, Jena, Germany; ^2^ Department of Horticulture, College of Agriculture, Isfahan University of Technology, Isfahan, Iran

**Keywords:** thyme species, terpenoids, phenolics, phytohormones, abscisic acid, gene expression, drought stress, LC-MS/MS

## Abstract

Thyme species, including *Thymus vulgaris, T. kotschyanus* (drought-tolerant) and *T. serpyllum* (drought-sensitive), are valuable medicinal herbs. They are often grown in arid regions and are increasingly suffering from water stress due to climate change. Here, we analyzed the metabolome and expression of selected genes in leaves of these species under drought stress with and without treatment with the phytohormone abscisic acid (ABA). Among the terpenes, dominant metabolites in thyme, thymol was the most important terpenoid component, followed by thymoquinone, carvacrol and p-cymene in all three species. Drought stress reduced terpene concentrations, while moderate ABA levels increased them. *T. kotschyanus* showed the highest concentrations of thymol and carvacrol after combined treatment with drought and ABA. Metabolite accumulation was partially correlated with genes related to terpenoid biosynthesis. The combined treatment of drought stress and ABA resulted in a significant reduction of the stress hormone jasmonic acid and an increase of its biosynthetic precursor, OPDA (cis-12-oxophytodienoic acid), in all species. The present research results indicate that ABA treatment at moderate concentrations could be used as a measure to increase the production of some pharmaceutically active phenolic monoterpenes in *T. vulgaris, T. serpyllum* and *T. kotschyanus* and increase the stress resistance of the plants.

## 1 Introduction

Medicinal plants are a key part in the pharmaceutical, health, and food industries. The Lamiaceae family is the largest plant family studied in pharmaceuticals, and its therapeutic significance stems from its essences (Askary et al., 2023). *Thymus* is one of the most popular medicinal genera and widely used in several industries, including food, cosmetics, and medicines (Mahdavi et al., 2020). It is commercially grown in the Mediterranean and middle East regions, with 215 species belonging to the Lamiaceae family (Rahimi et al., 2023).

Thyme species are perennial herbs that produce monoterpenes, sesquiterpenes, and phenolic compounds such as flavonoids and coumarins, which are important sources for treating different disorders. They show e.g. antitumor, antibacterial and antifungal activity and they are active against respiratory diseases (Patil et al., 2021). Rosmarinic acid is the most abundant phenolic acids in *T. vulgaris*, while thymol and carvacrol are the most common terpenoid components in its essential oils (Kianersi et al., 2021; Tohidi et al., 2017). These two terpenoid substances are derived from the methyl erythritol phosphate (MEP) pathway, which produces dimethylallyl diphosphate (DMADP) and isopentenyl diphosphate (IDP), which are subsequently used to eventually biosynthesize thymol and carvacrol as phenol monoterpenes (Dudareva et al., 2005). Geranyl diphosphate synthase (GDS) plays a crucial role in this metabolic pathway. It can catalyze the condensation of IDP and DMADP to geranyl diphosphate (GDP), the universal monoterpene precursor (Lichtenthaler, 1999). Following that, γ-terpinene synthase (TPS), a monoterpene synthase, generates γ-terpinene by cyclizing GDP. Furthermore, cytochrome P450 (CYP) monooxygenases *CYP71D178, CYP71D180*, and *CYP71D181* are involved in further alteration of the γ-terpinene skeleton to form thymol and carvacrol but only *CYP71D178* produces thymol, while *CYP71D180, CYP71D181*, and *CYP71D182* biosynthesize carvacrol (Kianersi et al., 2021; Krause et al., 2021; Tohidi et al., 2020).

The amounts of the different metabolites depend on the species (Yang et al., 2024), and also on environmental factors, in particular the availability of water (Arpanahi et al., 2020; Pant et al., 2021). Previous studies have shown that under extreme drought stress conditions, *T. kotschyanus* can grow and survive better than *T. vulgaris* (Ashrafi et al., 2018; Mohammadi et al., 2019). Thyme species have a high variation regarding drought tolerance and this might be attributed to differences in primary and secondary metabolite regulations (Ashrafi et al., 2022). Tolerant plants sometimes accumulate fewer metabolites than sensitive plants, despite producing more osmolytes (Mansinhos et al., 2022). Drought not only affects secondary metabolism but also phytohormone levels. In particular, it is well-known that, under drought or salinity, the concentration of abscisic acid (ABA) significantly increases, stimulating stomatal closure, adapting physiological responses, and causing alterations in gene expression (Jogawat et al., 2021). ABA is essential for plant resistance and adaptability to numerous abiotic stressors. In addition, ABA regulates reactions to environmental stresses, including heat, cold, salt, drought, and excessive levels of radiation (Liu et al., 2022). It has also been reported, that the external application of ABA improves the growth of crops such as lavender (Safari et al., 2024) and mugwort (Zehra et al., 2020) under abiotic stress conditions. Nowadays, there is a growing interest for using phytohormones to enhance the activity of antioxidant systems and improve plant tolerance to drought stress (Raza et al., 2022; Youssef et al., 2023). However, knowledge on the relationship between drought stress, the amounts of aromatic and medicinal compounds and ABA treatment in differentially drought-tolerant thyme species is limited.

Therefore, the research objectives were 1) to characterize and compare the phenolic compounds and terpenoid compositions, 2) to assess the expression of relevant genes for thymol and carvacrol biosynthesis, and to measure the stress-related phytohormone in drought tolerant thyme species, *T. kotschyanus*, a drought sensitive species, *T. serpyllum*, and of *T. vulgaris*, under drought stress with application of foliar spraying of ABA.

## 2 Materials and methods

### 2.1 Plant material and growth conditions

The seeds of three different thyme species, viz. *T. serpyllum*, *T. kotschyanus*, and *T. vulgaris*, were provided by the Research Institute of Forests and Rangeland in Tehran, Iran, and determined by Flora Iranica (Rechinger, 1963). Voucher specimens were deposited in the Research Institute of Forests and Rangeland Herbarium in Tehran, Iran. *T. vulgaris* is native to central and southern Europe, Africa and Asia. *T. serpyllum* to Central and Eastern Europe, mainly at higher altitudes, and the native range of *T. kotschyanus* is South and East Turkey to Iran.

The seeds germinated in a seedling plastic tray with a 1:1 blend of peat moss and coco peat. The trays were placed in a growing chamber (York) equipped with LED NS1 lights (Vayola, Norway) in December 2022. After two months, the seedlings were transplanted into plastic pots (10 cm in diameter and 8 cm in height, with five seedlings per pot). The pots growth media was made up of 3:2:1:0.5:0.5 ratios of sieved soil, sand-clay granule mix, perlite, vermiculite, and leccaton. Plants were kept at 22°C, and growth chamber settings included a 16-hour photoperiod, 35-50% relative humidity, and 180 μmol m^-2^, mimicking day-light conditions.

### 2.2 Treatment

All plots were irrigated on a daily basis for two months, after which the first foliar sprays of ABA (0, 25, and 50µM) (ABA0, ABA25, and ABA50, respectively) were carried out on the leaves of plants till runoff. ABA concentrations were chosen according to (Ghassemi et al., 2017; Safari et al., 2024; Youssef et al., 2023; Zehra et al., 2020). ABA (*cis, trans* abscisic acid, Sigma-Aldrich, Steinheim, Germany) was dissolved in ethanol, further diluted with distilled water, and then applied four times at one-week intervals. The treatment without ABA consisted 5-10 mL of ethanol mixed with distilled water. Drought stress was imposed one week after the initial spraying of ABA at two irrigation levels (100% and 40%; C = control and D = drought stress, respectively) of field capacity (FC). To calculate the amount of water required per pot at each irrigation regime, at the beginning of the experiment the soil FC was measured using the weighing method (Pourmeidani et al., 2017). First, the pots were filled with soil and thoroughly irrigated to achieve saturated soil. To prevent evaporation, the pot was covered with plastic and after 24 hours, pots were weighed every two hours. When the weight of the pots was fixed, the soil sample from each pot was weighed. The percentage of water in the soil under FC conditions was determined by the following equation:


$$\begin{array}{c} \text{Soil water content }(\%)\\ =(\text{soil fresh weight}-\text{soil dry weight}/\text{soil dry weight})\times 100 \end{array}$$

After subtracting the weight of the pot and the dry soil, the amount of water held under FC conditions was calculated, and different irrigation treatments (100% and 40% FC) were calculated accordingly. To this aim, the pots were weighed daily using the prescribed and calculated weight for each treatment, and the appropriate amount of water was added to each pot. Sampling was done one week following the last spraying. Both treated and untreated plants were frozen in liquid nitrogen and stored at -80°C until further analysis. This experiment solely examined vegetative development in thyme species.

### 2.3 RNA extraction and cDNA synthesis

The total RNA of thyme species leaves was extracted from 100 mg of powdered leaf using the InviTrapR Spin Plant RNA Mini Kit (50) (Invitek Molecular) according to the manufacturer’s instructions, followed by on-column DNA digestion with DNaseI (TURBO DNA-freeTM kit). RNA concentration, purity, and quality were measured with a spectrophotometer (NanoDrop, 2000c; Thermo Scientific). The RevertAid First Strand cDNA Synthesis Kit (Thermo Scientific, Schwerte, Germany) was used to synthesize cDNA from 900 ng/µl RNA. Oligo (dT) 18 primers (**Supplementary Table S1**) were used in accordance with manufacturer recommendations.

### 2.4 qRT - PCR and gene expression analysis

The normalized expression method (fold change) was used to analyze the relative transcript values of monoterpene synthase genes such as γ-terpinene synthase (*TPS2*), thymol synthase (*CYP71D178* and *CYP71D181*), and carvacrol synthase (*CYP71D180* and *CYP71D181*). Each sample had three biological and two technical replicates. Specific primers for qRT-PCR were constructed based on previously obtained conserved sequencing data of thyme species, and *EF1* was employed as a reference gene for normalization. Real-time PCR was performed using an iCycler (Bio-Rad, Hemel Hempstead, Germany) with SYBR green II (Agilent Technologies). The qRT-PCR settings were set as follows: an initial denaturation phase at 95°C for 3 minutes, followed by 40 cycles of denaturation at 95°C for 30 seconds, annealing at 57°C for 35 seconds, and a final elongation step at 72°C for 30 seconds. The primers used in the PCR and qRT-PCR procedures were gene-specific and are shown in **Supplementary Table S1**. Normalized raw Ct values were utilized to compare the results of control and treatment samples using the 2^-ΔΔCt^ (fold change) method, as previously described (Livak and Schmittgen, 2001).

### 2.5 Extraction of terpenes from thyme leaves

Young leaves from each species were sampled independently, frozen in liquid nitrogen, and ground to a fine powder using a mortar and pestle. A 50 mg plant powder was extracted with 400 μl hexane (containing 10 μg/ml of the internal standard nonyl acetate) in a 1.5 mL glass vial at room temperature for 1 hour. 50 μl of the hexane phase was transferred into a new glass vial for GC-MS analysis (Krause et al., 2021). The extract was subjected to gas chromatography using an Agilent 6890 series gas chromatograph (Agilent, Santa Clara, CA, USA), with 1 μL splitless injection and a flow rate of 2 mL min−1 using He as carrier. The components were separated on an Agilent DB-5MS column (30 m × 0.25 mm × 0.25 μm) with a temperature gradient of 45°C to 180°C at 6°C min^-1^, followed by an increase to 300°C at 100°C min^-1^. To identify compounds, the column outlet flow was connected to an Agilent 5973 quadrupole mass selective detector with an interface temperature of 270°C, quadrupole temperature of 150°C, source temperature of 230°C, and electron energy of 70 eV. For peak integration the following parameters were used initial peak width 0.08, initial threshold 18.0, integrator 5000-25000. The identity of each peak was determined by comparing its mass spectrum and retention time to authentic standards or spectra from reference libraries (NIST98 and Wiley 275) using MSD Chemstation. For compound quantification, the column outflow (H2 as carrier gas) was connected to a 300°C flame ionization detector. The amount of each chemical was calculated by comparing the peak area obtained to the peak area of the internal standard.

### 2.6 Analysis of phenolic compounds by LC-MS/MS

Approximately 50 mg of fresh thyme tissue was extracted and homogenized in 1.0 ml methanol containing 40 ng D6-JA (HPC Standards GmbH, Germany) and 10 ng trifluoro-methyl-cinnamic acid (Alfa Aesar, Karlsruhe, Germany) as internal standards. Samples were shaken on a horizontal shaker at room temperature for 10 minutes. The homogenate was blended for 30 minutes before being centrifuged at 13,000 rpm for 20 minutes at 4°C. The supernatant was then collected for analysis. Phenolic components in leaf extracts were analyzed according to (Ferlian et al., 2021) The Agilent 1260 infinity II LC system (Agilent Technologies, Waldbronn, Germany) was equipped with a Zorbax Eclipse XDB-C18 column (50 × 4.6 mm, 1.8 μm; Agilent Technologies, Waldbronn, Germany) with aqueous formic acid (0.05% (v/v)) and acetonitrile as mobile phases A and B. The mobile phase flow rate was 1.1 ml per minute. The elution profile was: 0-0.5 min, 5% B; 0.5-6.0 min, 5-37.4% B; 6.02-7.5 min, 80-100% B; 7.5-9.5 min, 100% B; and 9.52-12 min, 5% B. The column temperature was kept at 20°C. The LC system was connected to a QTRAP 6500 tandem mass spectrometer (SCIEX, Darmstadt, Germany) (with a turbospray ion source) that was set to negative ionization mode. The ion spray voltage was kept at -4500 eV, while the turbogas temperature was set to 650°C. The nebulizing gas was set at 60 psi, the curtain gas at 40 psi, the heating gas at 60 psi, and the collision gas at a medium setting. Multiple reaction monitoring (MRM) was utilized for quantification: analyte precursor ion (Q1) fragmentation into product ion (Q3) (see **Supplementary Table S2**). Data gathering and processing were carried out using Applied Biosystems’ Analyst 1.6.3 software. 5-caffeoyl-quinic acid, 3-caffeoyl-quinic acid, 4-caffeoyl-quinic acid, and sulfo-jasmonic acid were absolutely quantified using D6-JA as an internal standard and the response factors indicated in **Supplementary Table S2**. Coumaric acid, caffeic acid, and ferulic acid were absolutely quantified with trifluoro-methyl-cinnamic acid as an internal standard and the response factors provided in **Supplementary Table S2**. All other substances were relatively quantified, and the results are expressed as peak area per mg of tissue (Ferlian et al., 2021).

### 2.7 Analysis of phytohormones by LC-MS/MS

Approximately 50 mg of fresh thyme tissue was extracted and homogenized in 1.0 mL methanol containing 40 ng D4-SA (Santa Cruz Biotechnology, USA), 40 ng D6-JA (HPC Standards GmbH, Germany), 40 ng D6-ABA (Toronto Research Chemicals, Toronto, Canada), and 8 ng D6-JA-Ile (HPC Standards GmbH) as internal standards. Samples were shaken on a horizontal shaker at room temperature for 10 minutes. The homogenate was blended for 30 minutes before being centrifuged at 13,000 rpm for 20 minutes at 4°C. The supernatant was then collected for analysis. The phytohormone analysis was performed using LC-MS/MS (Heyer et al., 2018). Using an Agilent 1260 series HPLC system (Agilent Technologies) and a tandem mass spectrometer QTRAP 6500 (SCIEX, Darmstadt, Germany). Agilent Technologies’ Zorbax Eclipse XDB-C18 column (50 x 4.6 mm, 1.8 µm) was used for chromatographic separation. Water with 0.05% formic acid and acetonitrile were utilized as mobile phases A and B, respectively. The elution profile was as follows: 0-0.5 min, 10% B; 0.5-4.0 min, 10-90% B; 4.0-4.02 min, 90-100% B; 4.02-4.55 min, 100% B; and 4.51-7.0 min, 10% B. The flow rate was held constant at 1.1 ml/min, while the column temperature was kept at 25°C. The mass spectrometer was equipped with a Turbo spray ion source operated in negative ionization mode. The ion spray voltage remained at -4,500 eV. The turbogas temperature was set at 650°C. The nebulizing gas was set to 60 psi, the curtain gas at 40 psi, the heating gas at 60 psi, and the collision gas at “medium”. The mass spectrometer was operated in multiple reaction monitoring (MRM) mode; **Supplementary Table S3** contains information on the instrument parameters and response factors for quantification. Because we discovered that both the D6-labeled JA and D6-labeled JA-Ile standards (HPC Standards GmbH, Cunnersdorf, Germany) contained 40% of the corresponding D5-labeled compounds, the sum of the peak areas of D5- and D6-compounds was used for quantification.

### 2.8 Statistical analysis

The investigation was conducted as a factorial experiment using a completely randomized design with three replications. SAS version 9.4 (SAS Institute Inc., Cary, NC, USA) was used to analyze all parameter data by ANOVA (CRD). Means were compared using the LSD test to detect significant differences (p ≤ 0.05). Excel, JMP, and Statgraphics 16 software were used to analyze the data and draw the figures. Mean values are shown with their standard errors.

## 3 Results

### 3.1 Effects of drought and different ABA concentrations on terpenoid contents in different *Thymus* species

**Figure 1:**
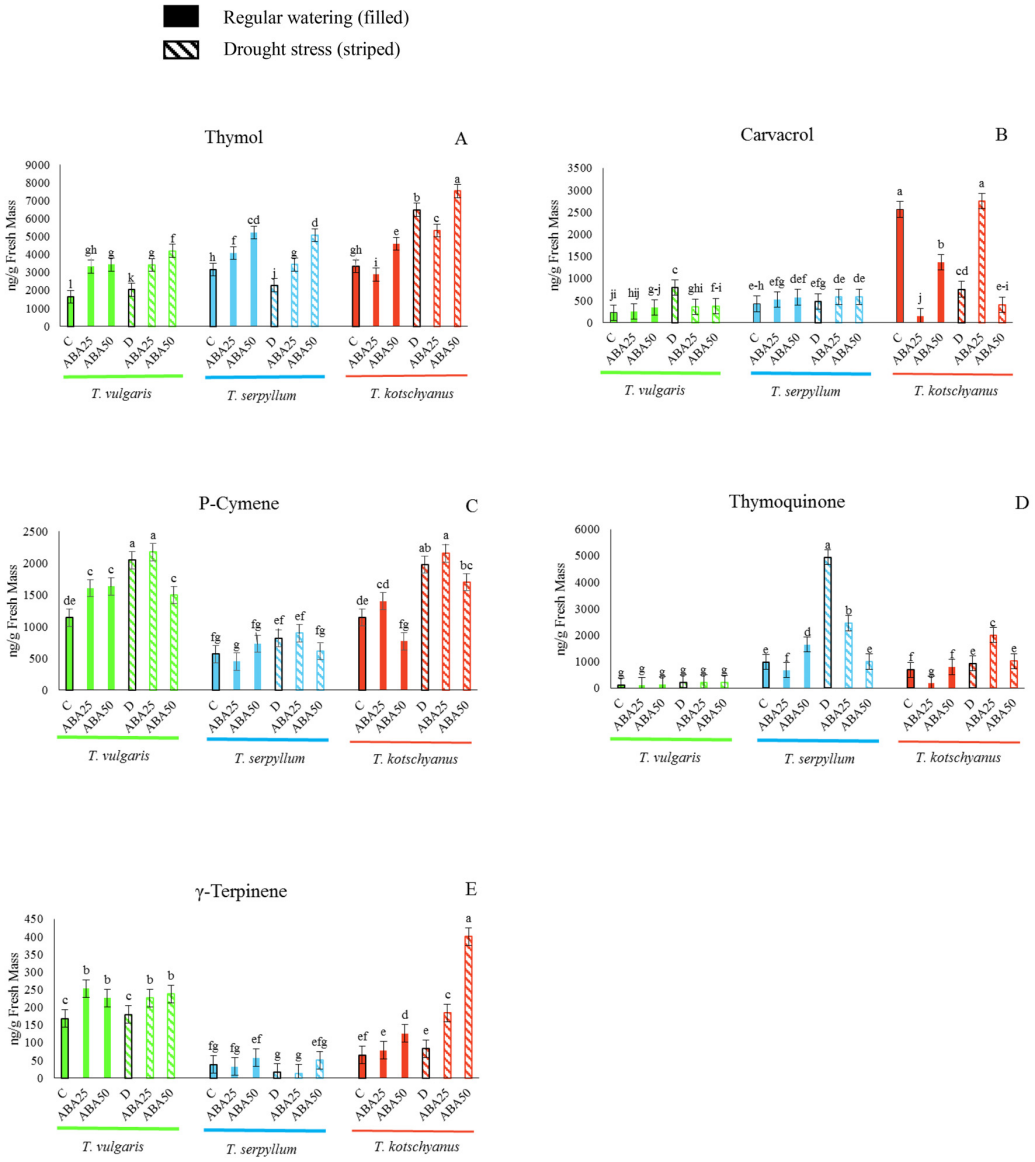
The major terpenoid compounds (**A**, thymol; **B**, carvacrol; **C**, *p*-cymene; **D**, thymoquinone; **E**, γ-terpinene) are differentially enriched in *T. vulgaris* (green), *T. serpyllum* (blue) and *T. kotschyanus* (red) and the contents are affected by different irrigation regimes (C, control or regular watering (filled bars), D, drought stress (striped bars)) and ABA (0, 25, 50 μM) treatment. Data shown are mean ± SE, n = 3, different letters indicate significant differences at *p* ≤ 0.05 (LSD test).

To determine how drought and ABA treatments affect the major terpenoid compounds in the three thyme species, the amounts of thymol, carvacrol, *p*-cymene, thymoquinone, and γ-terpinene in leaves were analyzed after the treatments (**Figure 1**). The three species differ substantially in the concentrations of the main terpenoids that accumulate in leaves under control conditions and show different responses to treatments. Under control conditions *T. vulgaris* is characterized by high levels of *p*-cymene and γ-terpinene, while *T. kotschyanus* shows significantly higher levels of carvacrol and, similar to *T. serpyllum*, the levels of thymol were significantly higher than for *T. vulgaris*. High concentrations of carvacrol were only found in *T. kotschyanus*. Drought stress increased the content of thymol, carvacrol, *p*-cymene, thymoquinone, and γ-terpinene. ABA treatments increased thymol, *p*-cymene, and γ-terpinene levels, while carvacrol and thymoquinone levels decreased (**Figure 1**). Thymol content increased in all three species as soil water content decreased from 100% to 40% FC and increased significantly with ABA concentration (**Figure 1A**). A control treatment and ABA25 × drought stress led to a significant increase in carvacrol levels in *T. kotschyanus* (**Figure 1B**), while in the other two species a pronounced change in carvacrol levels was only observed after drought treatment without ABA application. The *p*-cymene content increased when soil water content decreased from 100% to 40% FC in all three species (**Figure 1C**). Drought × ABA25 also led to a significant increase in *p*-cymene content in all three species, while higher ABA concentrations led to a decrease although the levels were still higher than in the controls (**Figure 1C**). Thymoquinone levels in *T. vulgaris* were negligible and unaffected by treatment, while ABA25 resulted in lower content in the other two species compared to controls, and drought and drought × ABA25 treatment resulted in a significant increase in thymoquinone levels in *T. serpyllum* and *T. Kotschyanus*, respectively (**Figure 1D**). The γ-terpinene levels increased significantly in *T. kotschyanus* and *T. vulgaris* by ABA treatment, while *T. serpyllum* showed the highest γ-terpinene content under control conditions (**Figure 1E**).

**Figure 2:**
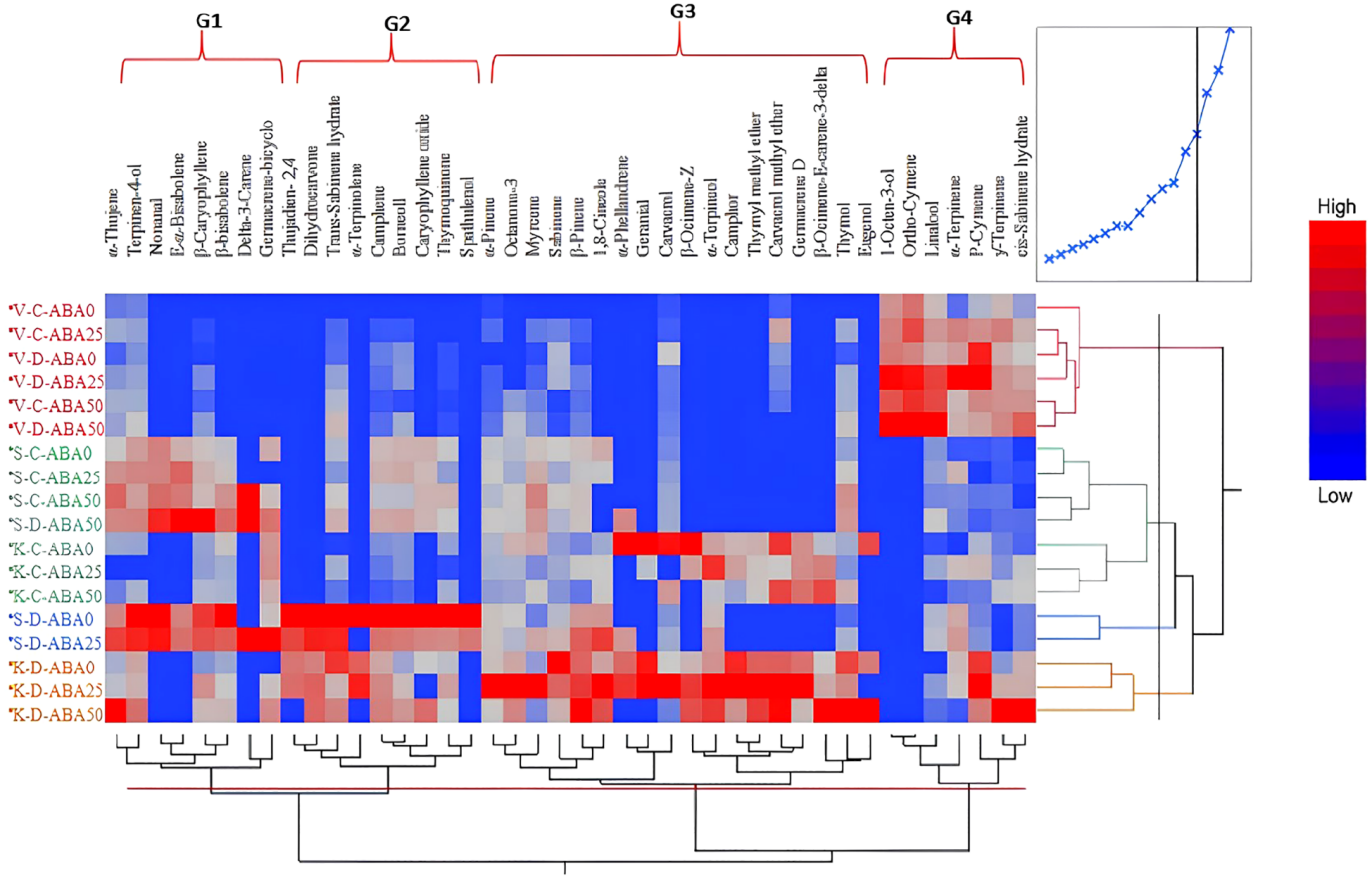
Hierarchically clustered heat map of different terpenoid compounds produced by three thyme species (V, *T. vulgaris*; S, *T. serpyllum*; K, *T. kotschyanus*) under different irrigation regimes (C, control or regular watering, D, drought stress) and ABA (0, 25, 50 μM) foliar spraying. The compounds separated into four groups. Different colors indicate the relative abundance of each metabolite.

A total of 42 compounds were identified in this experiment. An analysis using a heat map reveals distinct clusters, with the heat map’s colors demonstrating that the species vary in their levels of effective chemical production under both stress and non-stress conditions (**Figure 2**). Drought and ABA treatment affected metabolites across the four clusters, but with different changing patterns. *T. vulgaris* was categorized in the first cluster under both irrigation regimes and different ABA treatments. However, the production of compounds increased under drought conditions and the simultaneous application of ABA50. *T. serpyllum* and *T. kotschyanus* were grouped in the same cluster under non-stress conditions, but independent of the presence of ABA. The third cluster includes *T. serpyllum* under drought stress and ABA25. Finally, the fourth cluster includes *T. kotschyanus* under drought stress and different ABA treatments. In addition to species clustering, the heat map analysis identified four groups of terpenoid compounds (G1 – G4) that cluster together, indicating similar regulation of their accumulation (**Figure 2**).

Given the above analytical results, we used each compound in the terpenoids as a categorical variable and the proportion of compound content as a continuous variable to examine the degree of variation among the 42 main components. The variation in the levels of 38 identified compounds in the three thyme species under the different treatments ranged from 0 to 1000 ng/g FM. Thymol proved to be the most variable of all components (0-7541.4 ng/g DM), followed by thymoquinone (0-4939.13 ng/g FM), carvacrol (0-2749.65 ng/g FM), and *p*-cymene (0-2172.98 ng/g FM) (**Supplementary Figure S1**).

Overall, the results revealed a statistically significant relationship between water stress and ABA foliar sprays on the chemical terpenoid composition in thyme species (**Supplementary Table S4**). Under drought stress, often the amounts of terpenoids increased in proportion to the concentration of ABA.

### 3.2 Effects of drought and different ABA concentrations on the expression of selected thymol/carvacrol biosynthetic pathway genes in different *Thymus* species

**Figure 3:**
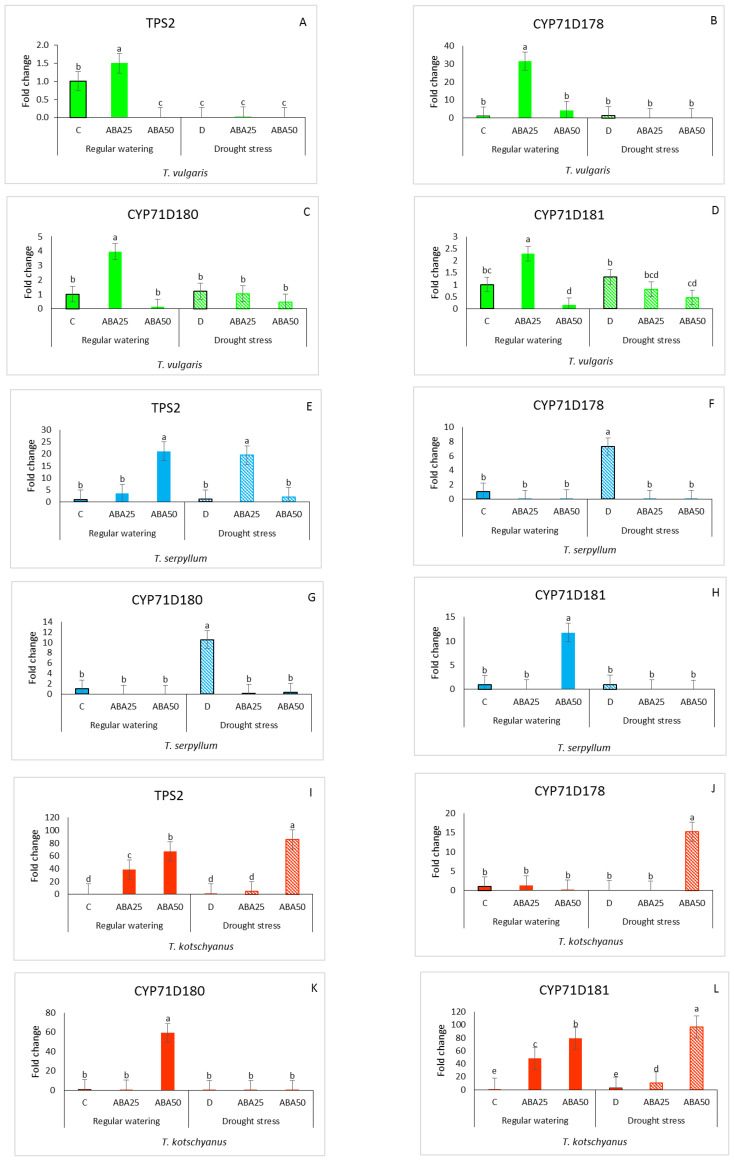
Relative expression of genes (*TPS2, CYP71D178, CYP71D180*, and *CYP71D181*) involved in terpenoid biosynthesis in *T. vulgaris, T. serpyllum* and *T. kotschyanus* leaves treated with different treatments of ABA (0, 25, 50 μM) and irrigation regimes (C = control or regular watering (filled bars), D = drought stress (striped bars)) **(A–L)**. Data shown are mean ± SE, n = 3, different letters indicate significant differences at *p* ≤ 0.05 (LSD test).

The relative gene expression of key biosynthetic enzymes such as *TPS2, CYP71D178, CYP71D180*, and *CYP71D181*, respectively, was studied to examine how the thymol/carvacrol biosynthesis genes might be altered in the leaves of *T. vulgaris*, *T. serpyllum*, and *T. kotschyanus* (**Figure 3**). In *T. vulgaris*, significant differences were observed in all tested genes in response to ABA25, with values 1.5, 31.5, 3.9, and 2.3 - fold higher than those of the control. In contrast, the effect decreased after treatment with ABA50. Drought stress with or without ABA had almost no significant effect on the gene expression, except for *TPS2* where drought caused no gene expression (**Figures 3A–D**). In *T. serpyllum*, irrigated conditions × ABA50 significantly increased the expression of *TPS2* and *CYP71D181* genes (21.08 and 11.79-fold higher than the control) (**Figures 3E, H**). In drought-treated leaves, *CYP71D178* and *CYP71D180* transcripts were significantly elevated, showing increases of 7.3 and 10.5-fold, respectively (**Figures 3F, G**). For *TPS2*, drought × ABA25 caused the highest expression level (**Figures 3E, H**). In *T. kotschyanus*, the application of ABA in watered plants resulted in a significant increase in mRNA levels of *TPS2* and *CYP71D181* at concentrations of 25 and 50 µM, with increases of 38.6 and 67.2-fold, and 48.5 and 79.4-fold, respectively. The expression of the *CYP71D180* gene was increased (59.17-fold) at ABA50 (**Figures 3I–L**). Under drought stress, the application of an additional ABA50 treatment led to a significant increase in gene expression of all tested genes (*TPS2, CYP71D178*, and *CYP71D181*) with fold changes of 85.5, 15.2, and 96.6, respectively, except for *CYP71D180* (**Figures 3I–L**).

### 3.3 Correlations between the main terpenoid components and gene expression

**Figure 4:**
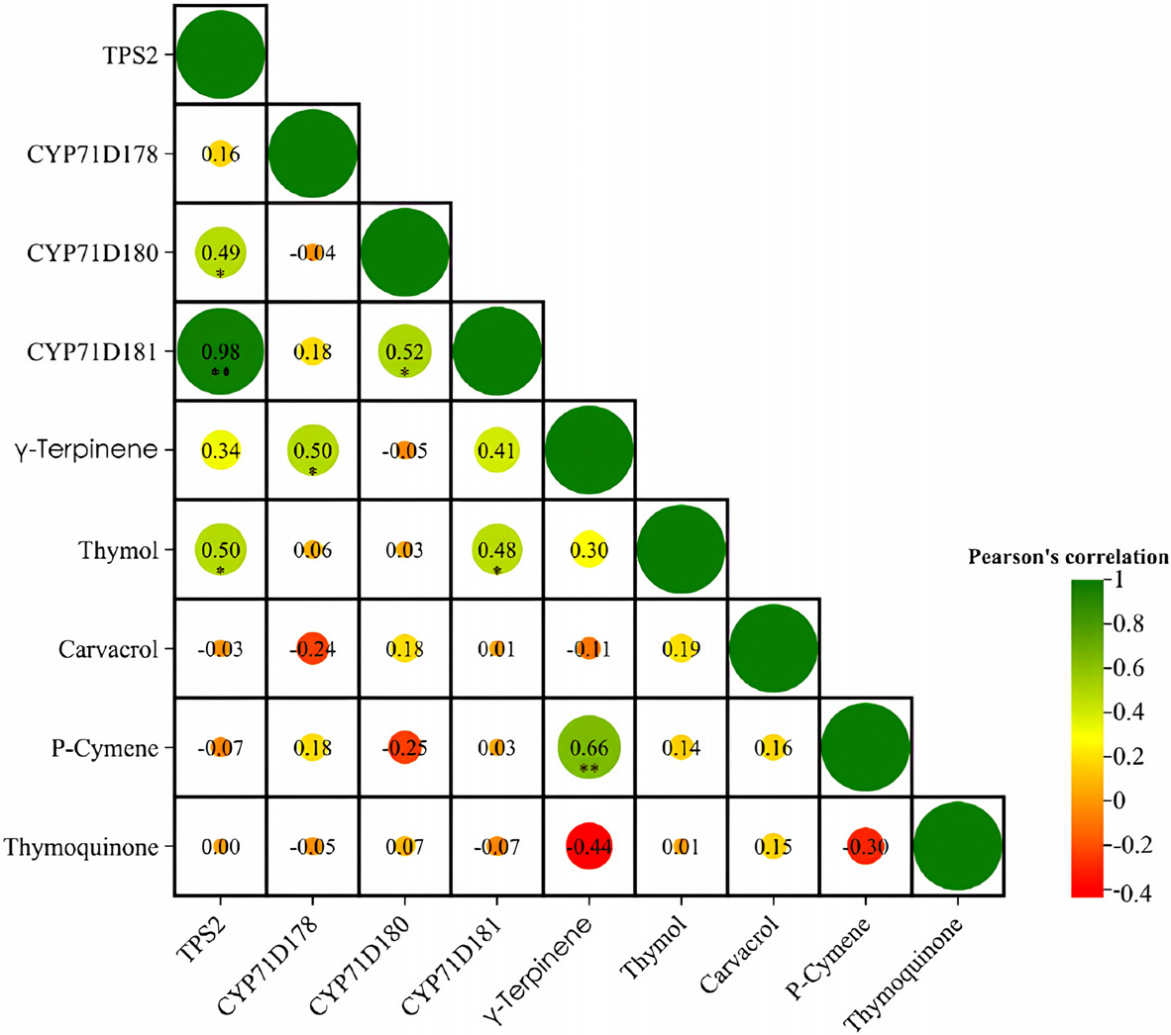
Pearson’s correlation coefficients between the main terpenoid components (thymol, carvacrol, *p*-cymene, thymoquinone, γ-terpinene) and gene expression (*TPS2, CYP71D178, CYP71D180*, and *CYP71D181*) on studied *Thymus* species (*T. vulgaris, T. serpyllum* and *T. kotschyanus*) affected by different irrigation regimes and ABA treatments. * Correlation is significant at the *p* ≤ 0.05 level. ** Correlation is significant at the *p* ≤ 0.01 level.

Pearson’s correlation analysis was performed to determine any relationships between the major terpenoid components and relative transcript levels (**Figure 4**). Among the investigated genes, *TPS2* and *CYP71D181* had the strongest correlation coefficient (*r* = 0.97**), followed by *CYP71D180* and *CYP71D181* (*r* = 0.52*) and *TPS2* and *CYP71D180* (*r* = 0.49*). Among the terpenoids, there was a strong correlation between γ-terpinene and *p*-cymene (*r* = 0.65**). As for the correlations between terpenoids and the biosynthesis genes, there was a clear correlation between thymol and both *TPS2* (*r* = 0.49*) and *CYP71D181* (*r* = 0.48*), respectively, as well as between γ-terpinene and *CYP71D178* (*r* = 0.49*). The strongest negative relations were found between thymoquinone and γ-terpinene (*r* = -0.4) and *p*-cymene (*r* = -0.3), respectively, followed by *CYP71D180* and *p*-cymene (*r* = -0.25) (**Figure 4**).

### 3.4 Effects of drought and different ABA concentrations on the phenolic compounds contents in different *Thymus* species

In addition to terpenoids, the phenolic compounds of *T. vulgaris*, *T. serpyllum*, and *T. kotschyanus* leaves were examined under drought stress and different ABA concentrations. Eighteen compounds were identified in the analysis (**Supplementary Tables S5A–C**), including ten different phenolic acids (*p*-coumaric acid, caffeic acid, ferulic acid, rosmarinic acid, rosmarinic acid glucoside, quinic acid, salvianolic acid, chlorogenic acids (5-caffeoylquinic acid, 5CQA; 4-caffeoylquinic acid (4QCA); and 3-caffeoylquinic acid (3QCA)), citric acid, four flavonoids (apigenin-6,8-di-C-glucoside, apigenin-glucuronide, luteolin-glucuronide, and quercetin), and three unknown compounds (555-359, 307-263, and 399-161). Both ABA and drought treatments significantly altered the polyphenol content in the three thyme species. Rosmarinic acid had the largest peak area of the phenolic components measured among all three thyme species, with no significant differences observed between treatments (**Supplementary Tables S5A–C**). Drought stress significantly reduced the levels of caffeic acid and 5CQA in *T. vulgaris*, an effect that was mitigated by ABA treatment. ABA and drought led to a reduction of citric acid levels, whereas ABA treatment alone increased the levels of apigenin-6,8-di-C-glucoside and quercetin in plants that were sufficiently irrigated. All other compounds did not show any clear tendencies upon drought or ABA treatment (**Supplementary Tables S5A–C**). In *T. serpyllum*, levels of quinic and salvianolic acids increased after drought stress but were reduced due to further ABA treatments. In contrast to *T. vulgaris*, the citric acid levels increased after ABA treatment, whereas apigenin-6,8-di-C-glucoside and rosmarinic acid glucoside decreased in watered plants. The unknown compound 307-263 showed a significant decrease after ABA treatment (**Supplementary Tables S5A–C**). In *T. kotschyanus*, this latter effect was also detected as well as the apigenin-6,8-di-C-glucoside and rosmarinic acid glucoside decrease upon ABA treatment. Quinic acid concentrations increased after treatment with drought and ABA treatment. The other tested compounds showed no clear trends after drought or ABA treatment (**Supplementary Tables S5A–C**). Notably, quercetin was only detectable in *T. vulgaris*. In the other two species, ABA, or even drought in *T. kotschyanus*, led to the elimination of the accumulation of this flavonoid (**Supplementary Tables S5A–C**).

### 3.5 Effects of drought and different ABA concentrations on the phytohormone contents in different *Thymus* species

Phytohormones mediate stress responses in plants. Consequently, the present analysis will encompass both the secondary metabolites in the three *Thymus* species and the stress-relevant phytohormones (**Supplementary Table S6**). *T. vulgaris* showed the lowest concentration of SA in comparison to the other two species, while *T. kotschyanus* displayed the highest levels, which further increased under drought stress. Drought stress decreased SA content in *T. vulgaris*, while in *T. kotschyanus* SA content increased; however, no significant differences were observed between treatments within each species. The SA-Gluc levels were observed to reflect the distribution of SA and remained largely unaffeted by the different treatments. For JA, significant differences were observed among the species, drought stress, and ABA treatment. While *T. vulgaris* and T*. kotschyanus* showed comparable values, in *T. serpyllum* the JA content was 10 to 20-fold lower. The JA degradation metabolites (OH-JA, sulfo-JA) showed the same distribution among the species. In addition, the decrease of JA in watered plants upon ABA treatment was not detected in *T. serpyllum*. Particularly, these findings were not observed for JA-Ile. Here, for all three species the levels detected were very similar. This is applicable to OH-JA-Ile; however, COOH-JA-Ile was not detected in *T. serpyllum*. The biosynthetic jasmonate precursor, *cis*-OPDA, did not show any clear trends.

**Figure 5:**
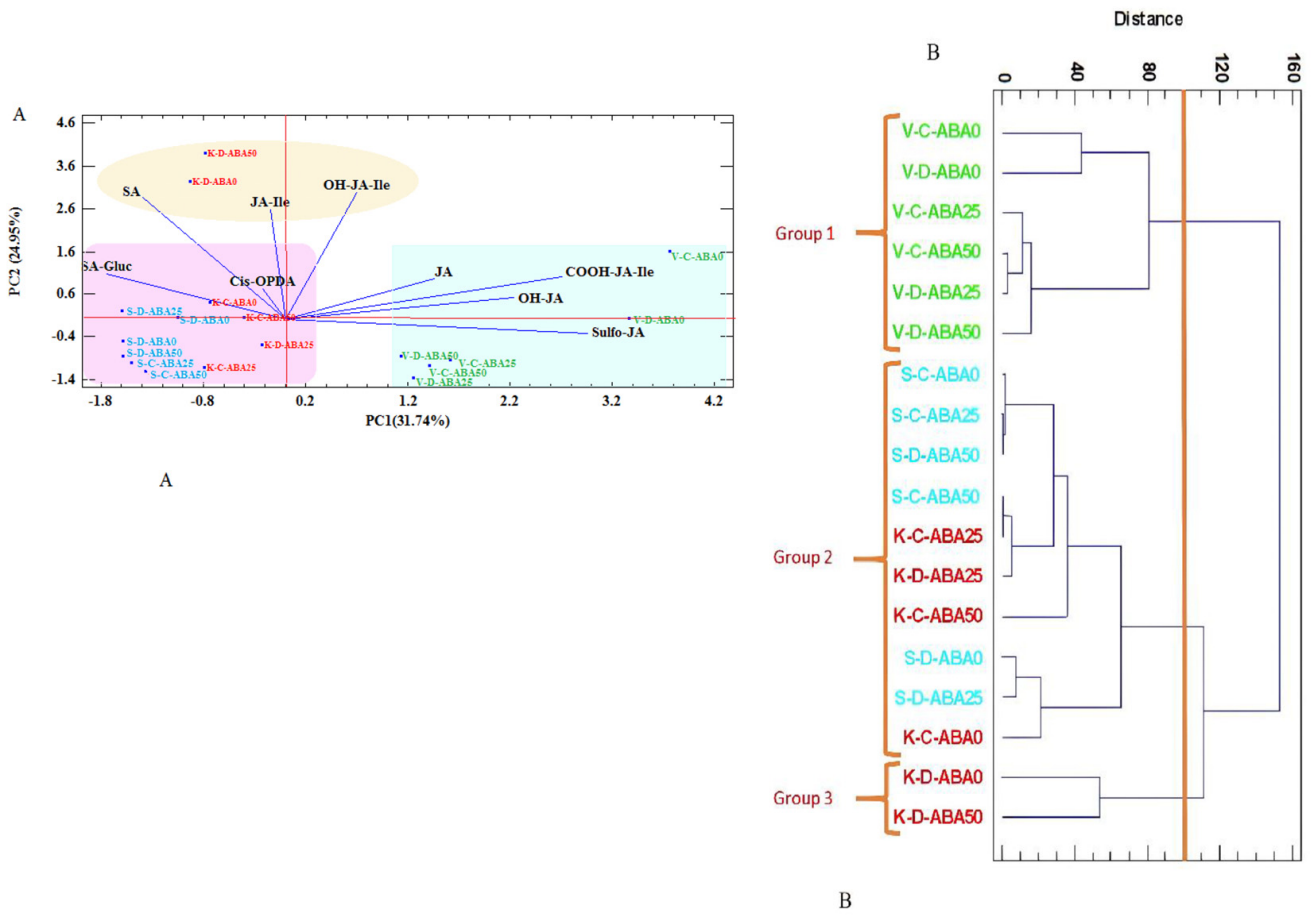
Principal Component Analysis (PCA) **(A)** and Cluster Analysis (CA) **(B)** for stress-related phytohormones in thyme species (V= *T. vulgaris* (green), S= *T. serpyllum* (blue), K= *T. kotschyanus* (red)) treated with different irrigation regimes (C= control or regular watering, D= drought stress) and ABA (0, 25, 50 μM) treatment. SA (salicylic acid), SA-Gluc (salicylic acid-β-D-glucoside), *Cis*-OPDA (12-oxophytodienoic acid), JA (jasmonic acid), JA-Ile (jasmonoyl-isoleucine conjugate), OH-JA-Ile (hydroxyjasmonoyl-isoleucine conjugate), OH-JA (hydroxyjasmonic acid), COOH-JA-Ile (carboxy jasmonoyl-isoleucine conjugate), Sulfo-JA (sulfated jasmonic acid), point labels: number of complete cases, vectors: data variables.

Next, cluster analysis and principal component analysis (PCA) were conducted to evaluate the phytohormone differences among the samples and treatments, as well as the variability within the groups (**Figure 5**). According to PCA, the first three principal components (PC1, PC2, and PC3) explained the majority of the variation (69.88%) (**Supplementary Table S7**). The PCA results revealed that the first and second components explained 56.69% of the overall variation. The first PC (PC1) explained 31.74% of the total variation and had a high positive correlation with sulfo-JA (0.55) and COOH-JA-Ile (0.5) contents, while demonstrating a negative correlation with SA-Gluc (-0.32) and SA (-0.25) levels. PC2 explained 24.95% of total variance, with a positive correlation with OH-JA-Ile (0.56) and SA (0.54) and a negative correlation with sulfo-JA (-0.06) (**Figure 5**) (**Supplementary Table S7**).

PCA analysis categorized the species into three distinct groups. Group 1, which included *T. vulgaris* under both irrigation regimes, showed high amounts of JA, OH-JA, COOH-JA-Ile, and sulfo-JA. In contrast, Group 2, which included *T. kotschyanus* and *T. serpyllum* species, had a high SA-Gluc and cis-OPDA content. Group 3 (drought stressed *T. kotschyanus* without and with high ABA treatment) had components including SA, JA-Ile, and OH-JA-Ile (**Figure 5A**). The cluster analysis also confirmed these findings (**Figure 5**).

## 4 Discussion

Drought stress significantly threatens agricultural production. This concerns not only crop plants but also medicinal herbs, as the yield of essential oils can be significantly impacted (Askary et al., 2018). Here, drought tolerance showed significant variations in the content of the predominant terpenoids under well-irrigated conditions as well as in their response to drought stress; however, no consistent pattern was observed. Thymol, *p*-cymene, and γ-terpinene levels in *T. vulgaris* and *T. kotschyanus* increased after drought stress, whereas the drought-susceptible species *T. serpyllum* showed significantly lower levels. These findings were in line with the previous researches indicating increased concentrations of thymol, carvacrol, and linalool in *T. kotschyanus* and *T. vulgaris* (Askary et al., 2018; Mohammadi et al., 2019). In the current study, although *T. kotschyanus* showed the highest carvacrol content under control conditions, the contents strongly declined after drought treatment. Interestingly, the terpenoid content and composition of *T. vulgaris* groups clearly apart from the two other species with octenol, linalool, and ortho-cymene being significantly induced by drought stress, while the drought-susceptible and the drought-tolerant lines are less clearly separated. *T. serpyllum* is characterized by increased levels of nonanal, bisabolene and beta-caryophyllene after drought stress, while *T. kotschyanus* showed the strongest increase in 15 terpenoids after combined drought and ABA treatment without a specific-drought-related effect.

Based on the previous researches, ABA foliar spraying was used to investigate if ABA treatment increases drought tolerance without impeding the production of essential oils; ideally even enhancing the secondary metabolites production. Exogenous ABA treatment has been demonstrated to protect *Artemisia annua* from drought stress by increasing antioxidant enzyme activity, which helps in the maintenance of leaf cell integrity and leads to enhanced artemisinin synthesis (Zehra et al., 2020). Additionally, foliar application of ABA enhanced the secondary metabolite content in *Carum copticum* compared to untreated control plants (Ghassemi-Golezani et al., 2018). In conclusion, ABA enhances the synthesis of many bioactive compounds and increases secondary metabolite accumulation, which helps to improve the medical uses of plants (Ghassemi et al., 2017). Consistent with these studies, an increase in key terpenoids (thymol, *p*-cymene, and γ-terpinene) was observed in all three species, whereas carvacrol levels either remained stable or decreased. This result might be explained with a different flux of metabolites in the biosynthetic pathway. It was already proposed that thymol and carvacrol are synthesized directly from γ-terpinene as a substrate, while *p*-cymene is created as a by-product of the substrate’s premature release from the active site (Crocoll et al., 2010). Furthermore, ABA treatment may preferentially enhance the production of thymol over carvacrol. In general, ABA foliar sprays could increase the percentages of most monoterpene components in two ways: (1) by directly or indirectly influencing isoprenoid metabolism; and (2) by increasing the pH of the chloroplast stroma following stomatal closure and consequently affecting the activity of the enzyme that synthesizes monoterpenes (Ghassemi-Golezani et al., 2018).

A change in metabolite levels might be due to alterations in biosynthetic gene expression. This study investigated an early gene *TPS2*, and late genes *CYP71D178/180/181* in the monoterpenoid pathway, which all are involved in thymol and carvacrol biosynthesis (Kianersi et al., 2021; Tohidi et al., 2020). According to the present research findings, all of the genes investigated in thymol and carvacrol production pathways responded to ABA and drought. Treatments increased transcription levels, but the extent of the increase differed depending on the species and gene of interest. *TPS2* showed the largest increase in response to ABA and drought in all three species. The data demonstrate that *TPS2* is more affected than *CYP71D178, CYP71D180*, and *CYP71D181*, indicating that it plays an important role in thymol and carvacrol biosynthesis. Similar results were observed in a study conducted on thyme, and it was reported that the relative expression of these genes was influenced by MeJA, SA, and UV-C treatments. These elicitors and their effects on terpene production have already been reported from numerous plant species, including *Thymus* spp (Askary et al., 2018; Mohammadi et al., 2019; Kianersi et al., 2021; Amani Machiani et al., 2023; Abbasi et al., 2024), *Lavandula angustifolia* (Safari et al., 2024), and *T. kotschyanus* (Kamali et al., 2024).

On the other hand, there was an adverse correlation between *CYP71D181* expression and *p*-cymene levels. The lack of congruence between the transcriptional levels of the genes and their related compounds (less gene expression but more compound production) may be due to stress affecting enzyme activity or particular alterations in transcription and post-translational processes (Azimzadeh et al., 2023). The participation of *p*-cymene, an aromatic hydrocarbon, and the type of enzymes involved in aromatic ring formation remains unknown (Krause et al., 2021).

Similar to previous studies (Sarfaraz et al., 2021; Yang et al., 2024), it was observed that some compounds changed after drought stress and ABA treatment but the changes were species- and compound-specific resulting in highly variable patterns. This means that in all three *Thymus* species not only the levels of some phenolic compounds strongly differed among the species but also that their levels were affected differently by drought and foliar spray of ABA, indicating a species and compound-specific metabolite flux. In general, phenolic compounds are assumed to improve the antioxidant capacity and abiotic stress resistance (Parvin et al., 2022); but based on our results we cannot pinpoint which compound might have a protective role against drought.

Furthermore, drought stress led to significantly lower levels of the phytohormone jasmonic acid in all three species, while OPDA levels were significantly higher. This is consistent with the idea that drought stress inhibits the conversion of OPDA to JA, leading to an accumulation of OPDA, which may induce stomatal closure as response to water stress to reduce water loss (Savchenko et al., 2014). The intervention of JAs in drought stress has been reported in a number of plant species (Brossa et al., 2011; Pedranzani et al., 2007; Wahab et al., 2022). Interestingly, the general picture of the phytohormone response and the terpenoid pattern are highly similar. For both groups of compounds, *T. vulgaris* is clearly separate from the two other species. At the phytohormonal level, *T. vulgaris* is characterized by the presence of JA- and inactive JA-derivatives, while *T. serpyllum* and *T. kotschyanus* mostly group together and are determined by salicylic acid-β-D-glucoside accumulation. Further, drought-stressed *T. kotschyanus* leaves are characterized by the bioactive JA-Ile and SA. This correlation among the two groups of compounds suggest a close interaction between phytohormone levels and terpenoid production.

## 5 Conclusions

In this study, three *Thymus* species showed increased levels of major essential oil components after foliar spray with ABA, in particular at a lower concentration of 25 µM, while drought stress mostly led to a decrease of these compounds. The drought tolerant species *T. kotschyanus* showed particularly high levels of the terpenoids thymol, *p*-cymene, γ-terpinene, and carvacrol after combined drought stress and ABA treament. The increase only partially correlated with key genes of the terpenoid biosynthesis pathway and depended on the particular species. Phytohormone levels also differed among treatments and species, although across all species the combination of drought and ABA led to a significant reduction in JA and an increase in OPDA levels. The present research findings suggest that ABA treatment can help *T. vulgaris*, *T. serpyllum*, and *T. kotschyanus* producing more pharmaceutically active phenolic monoterpenes. An integrated investigation of metabolomics and transcriptomics in most of the thyme species combined with specific alterations of the biosynthetic pathway might open the way to understand and manipulate the regulatory mechanisms that control the production of pharmaceutically important compounds under environmental stress.

## Data Availability

The original contributions presented in the study are included in the article/**Supplementary Material**. Further inquiries can be directed to the corresponding authors.
