# Supplementary material for: Integrated metabolomics, transcriptomic, and phytohormonal analyses to study the effects of water stress and foliar abscisic acid application in Thymus species using LC-MS/MS
Source: Front Plant Sci. 2025 Mar 11;16:1557446. doi: 10.3389/fpls.2025.1557446 (PMC11933024; doi:10.3389/fpls.2025.1557446)
Supplement: Supplementary file 1 [file DataSheet1.docx]

Supplementary Material

# Supplementary Tables and Figures

## Supplementary Tables

**Supplementary Table 1.** Primers used for qRT-PCR analysis.

| Real-Time Primers | Sequences (5´ to 3´) | Tm°C | Reference |
| --- | --- | --- | --- |
| *Ef1 F*  *Ef1 R* | AGATCGGAAATGGTTATGCTC  GACCTCCTGTCAATCTTCGT | 55.9  57.3 | Ashrafi et al., 2022 |
| *TPS2 F*  *TPS2 R* | AACCTCGCCGAGAAACTCCC  AGCTGCAGTTCGTCGAGTGT | 61.4  59.4 | Tohidi et al., 2020 |
| *CYP71D178 F*  *CYP71D178 R* | CAAGGAATGACTGCTGCTGAC  TTGGATTGTGGATTGTTGGAACC | 59.8  58.9 | Crocoll, 2011 |
| *CYP71D180 F*  *CYP71D180 R* | GCAAAGAAGAATGCGAGGTC  GATTGAACGTGTCGGGATCT | 57.3  57.3 | Crocoll, 2011 |
| *CYP71D181 F*  *CYP71D181 R* | TACTGGAAAGACCCCGACAC  CGAACGGGATTAACTCGAAA | 59.4  55.3 | Crocoll, 2011 |

Ref:

Ashrafi, M., Azimi-Moqadam, M. R., MohseniFard, E., Shekari, F., Jafary, H., Moradi, P., et al. (2022). Physiological and molecular aspects of two *Thymus* species differently sensitive to drought stress. *BioTech*. 11(2), 8. https://doi.org/10.3390/biotech11020008

Crocoll, C. (2011). Biosynthesis of the phenolic monoterpenes, thymol and carvacrol, by terpene synthases and cytochrome P450s in oregano and thyme (Doctoral dissertation, Jena, Friedrich-Schiller-Universität Jena, Diss., 2011).

Tohidi, B., Rahimmalek, M., Arzani, A., and Trindade, H. (2020). Sequencing and variation of terpene synthase gene (TPS2) as the major gene in biosynthesis of thymol in different *Thymus* species. *Phytochemistry.* 169, 112126. https://doi.org/10.1016/j.phytochem.2019.112126

**Supplementary Table 2**. Details of analysis of phenolic compounds by LC-MS/MS [HPLC 1260 (Agilent Technologies)-QTRAP6500 (SCIEX)] in negative ionisation mode

| Q1 | Q3 | RT (min) | Compound | Internal std | RF | DP | EP | CE | CXP |
| --- | --- | --- | --- | --- | --- | --- | --- | --- | --- |
| 353.0 | 190.9 | 3.65 | 5-caffeoyl-quinic acid | D6-JA | 1.15 | -20 | -4 | -22 | -4 |
| 353.0 | 179.1 | 3.00 | 3-caffeoyl-quinic acid | D6-JA | 0.89 | -20 | -4 | -22 | -4 |
| 353.1 | 173.0 | 3.80 | 4-caffeoyl-quinic acid | D6-JA | 0.81 | -20 | -4 | -22 | -4 |
| 305.0 | 97.0 | 4.00 | Sulfo-Jasmonic acid | D6-JA | 6.00 | -20 | -5 | -55 | -2 |
| 163.0 | 118.9 | 4.70 | coumaric acid | TriFluoro-methyl-cinnamic acid | 2.56 | -20 | -8 | -20 | -5 |
| 179.0 | 134.9 | 4.00 | caffeic acid | TriFluoro-methyl-cinnamic acid | 1.62 | -20 | -8 | -22 | -5 |
| 193.1 | 133.9 | 5.00 | ferulic acid | TriFluoro-methyl-cinnamic acid | 7.77 | -20 | -8 | -22 | -5 |
| 359.0 | 161.0 | 5.70 | rosmarinic acid |  |  | -20 | -8 | -25 | -5 |
| 493.0 | 295.0 | 5.90 | salvianolic acid-295-1 |  |  | -20 | -8 | -25 | -5 |
| 493.0 | 295.0 | 6.10 | salvianolic acid-295-2 |  |  | -20 | -8 | -25 | -5 |
| 593.0 | 473.0 | 4.00 | apigenin-6,8-di-C-glucoside |  |  | -20 | -8 | -25 | -5 |
| 521.0 | 359.0 | 5.00 | rosmarinic acid glucoside |  |  | -20 | -8 | -25 | -5 |
| 461.0 | 285.0 | 5.20 | luteolin-glucuronide |  |  | -20 | -8 | -25 | -5 |
| 445.0 | 269.0 | 5.50 | apigenin-glucuronide |  |  | -20 | -8 | -25 | -5 |
| 537.0 | 493.0 | 6.00 | lithospermic acid A |  |  | -20 | -8 | -25 | -5 |
| 555.0 | 359.0 | 5.70 | 555-359 |  |  | -20 | -8 | -25 | -5 |
| 307.0 | 263.0 | 1.90 | 307-263 |  |  | -20 | -8 | -25 | -5 |
| 399.0 | 161.0 | 4.40 | 399-161 |  |  | -20 | -8 | -25 | -5 |
| 301.0 | 93.0 | 6.80 | quercetin |  |  | -20 | -8 | -46 | -5 |
| 191.0 | 111.0 | 0.70 | citric acid |  |  | -20 | -8 | -18 | -5 |
| 191.0 | 85.0 | 0.50 | quinic acid |  |  | -20 | -8 | -28 | -5 |
| 215.1 | 171.1 | 7.30 | TriFluoro-methyl-cinnamic acid |  |  | -20 | -8 | -18 | -4 |
| 215.0 | 59.0 | 7.20 | D6-JA |  |  | -20 | -9 | -24 | -2 |

**Supplementary Table 3**. Details of analysis of phytohormones by LC-MS/MS [HPLC 1260 (Agilent Technologies)-QTRAP6500 (SCIEX)] in negative ionisation mode

| Q1 | Q3 | RT (min) | Compound | Internal std | RF | DP | EP | CE | CXP |
| --- | --- | --- | --- | --- | --- | --- | --- | --- | --- |
| 136.93 | 93.00 | 3.3 | SA | D4-SA | 1.0 | -20 | -8 | -24 | -7 |
| 263.00 | 153.20 | 3.4 | ABA | D6-ABA | 1.0 | -20 | -12 | -22 | -2 |
| 209.07 | 59.00 | 3.6 | JA | D6-JA | 1.0 | -20 | -9 | -24 | -2 |
| 322.19 | 130.10 | 3.9 | JA-Ile | D6-JA-Ile | 1.0 | -50 | -4.5 | -30 | -4 |
| 290.90 | 165.10 | 4.6 | OPDA | D6-JA | 1.0 | -20 | -12 | -24 | -2 |
| 338.10 | 130.10 | 3 | OH-JA-Ile | D6-JA-Ile | 1.0 | -50 | -4.5 | -30 | -4 |
| 225.10 | 59.00 | 2.6 | OH-JA | D6-JA | 1.0 | -20 | -9 | -24 | -2 |
| 352.10 | 130.10 | 3 | COOH-JA-Ile | D6-JA-Ile | 1.0 | -50 | -4.5 | -30 | -4 |
| 140.93 | 97.00 | 3.3 | D4-SA |  |  | -20 | -8 | -24 | -7 |
| 269.00 | 159.20 | 3.4 | D6-ABA |  |  | -20 | -12 | -22 | -2 |
| 215.00 | 59.00 | 3.6 | D6-JA |  |  | -20 | -9 | -24 | -2 |
| 214.00 | 59.00 | 3.6 | D5-JA |  |  | -20 | -9 | -24 | -2 |
| 328.19 | 130.10 | 3.9 | D6-JA-Ile |  |  | -50 | -4.5 | -30 | -4 |
| 327.19 | 130.10 | 3.9 | D5-JA-Ile |  |  | -50 | -4.5 | -30 | -4 |

**Supplementary Table 4**. Analysis of variance of the data for the main terpenoids found in thyme species treated after application of different concentrations of ABA and drought treatment

|  |  | mean squares | | | | |
| --- | --- | --- | --- | --- | --- | --- |
|  | df | Thymol | Carvacrol | P-Cymene | Thymoquinone | γ-Terpinene |
| Species | 2 | 18533936.58** | 4634211.49** | 5229417.88** | 14429496.41** | 152651.26** |
| Drought | 1 | 11001633.81** | 72581.26** | 3259699.13** | 9741561.49** | 20165.78** |
| ABA | 2 | 16190858.01** | 321176.75** | 378676.62** | 1242138.79** | 38106.12** |
| Species ×Drought | 2 | 13785477.53** | 97725.6** | 469487.88** | 2924014.18** | 30223.02** |
| Species × ABA | 4 | 2544758.16** | 365670.8** | 130819.77** | 1787827.84** | 14008.83** |
| Drought× ABA | 2 | 344035.26** | 2440709.04** | 243657.51** | 3214811.07** | 10289.11** |
| Species ×Drought× ABA | 4 | 117235.22** | 2944197.72** | 154828.15** | 2995256.6** | 7928.12** |
| Error | 36 | 5336 | 4175.33 | 11792.55 | 1662.59 | 146.03 |
| CV |  | 1.8 | 8.61 | 8.37 | 4 | 8.9 |

ns, * and **: No significant and significant at *p* ≤ 0.05 and *p* ≤ 0.01, respectively.

**Supplementary Table 5A.** Phenolics content (ng/g FW) in thyme species after application of different concentrations of ABA and drought treatment.

| Species | Irrigation regimes(%fc) | ABA (µM) | Coumaric acid | Caffeic acid | Ferulic acid | Chlorogenic acid  (5-CQA) | Chlorogenic acid  (4-CQA) | Chlorogenic acid  (3-CQA) |
| --- | --- | --- | --- | --- | --- | --- | --- | --- |
| *T.vulgaris* | Regular watering | 0 | 546 ±166 | 58037±268 | 319±96 | 53305±2135 | 17823±3328 | 6925±58 |
|  |  | 25 | 449±62 | 43505±13561 | 213±122 | 106239±19797 | 11681±136 | 5222±252 |
|  |  | 50 | 173±5 | 51107±6260 | 248±11 | 25361±2905 | 19337±1592 | 10029±303 |
|  | Drought stress | 0 | 428±140 | 37242±2049 | 418±150 | 14151±2027 | 18149±2748 | 6785±2024 |
|  |  | 25 | 331±59 | 24360±7260 | 285±53 | 49631±19418 | 12123±80 | 7287±3106 |
|  |  | 50 | 472±22 | 43809±2833 | 422±24 | 8964±1449 | 24411±3239 | 9616±4492 |
| *T.serpyllum* | Regular watering | 0 | 179±43 | 19344±1330 | 430±79 | 962±188 | 20263±2039 | 4760±934 |
|  |  | 25 | 328±61 | 20093±280 | 477±66 | 268±39 | 11479±547 | 2781±232 |
|  |  | 50 | 271±60 | 27905±1787 | 352±26 | 1282±44 | 42360±8478 | 4141±588 |
|  | Drought stress | 0 | 421±80 | 20157±3217 | 399±20 | 3721±810 | 46485±199 | 11395±1416 |
|  |  | 25 | 491±17 | 34455±6102 | 551±172 | 1198±497 | 18148±12701 | 4251±3592 |
|  |  | 50 | 237±15 | 31204±3220 | 905±186 | 2446±435 | 23801±1916 | 5013±341 |
| *T.kotschyanus* | Regular watering | 0 | 502±40 | 25685±317 | 1533±18 | 1383±137 | 61513±6663 | 20511±4389 |
|  |  | 25 | 450±72 | 29624±3461 | 519±49 | 1621±299 | 128945±33205 | 23170±11637 |
|  |  | 50 | 617±38 | 30681±177 | 573±8 | 14699±3550 | 118060±1975 | 12816±256 |
|  | Drought stress | 0 | 699±16 | 23895±3704 | 1000±190 | 2348±246 | 134496±19210 | 39746±4690 |
|  |  | 25 | 651±179 | 26753±8799 | 1305±165 | 14687±2223 | 124168±32573 | 47638±6842 |
|  |  | 50 | 718±275 | 20142±1131 | 832±58 | 2100±122 | 126516±1311 | 23884±2745 |
| LSD 5% |  |  | 98.14 | 4842.99 | 100.38 | 6445.59 | 12124.63 | 3806.22 |

∗nd: non detected

**Supplementary Table 5B.** Phenolics content (peak area/mg FW) in thyme after application of different concentrations of ABA and drought treatment.

| Species | Irrigation regimes(%fc) | ABA (µM) | Rosmarinic acid | Quinic acid | Salvianolic acid | Rosmarinic  acid glucoside | Citric acid | Apigenin  6,8-di-C-glucoside |
| --- | --- | --- | --- | --- | --- | --- | --- | --- |
| *T. vulgaris* | Regular watering | 0 | 16614022±3243517 | 1001030±25825 | 21250±8933 | 198823±84344 | 2912741±292931 | 13286±2492 |
|  |  | 25 | 16602101±289432 | 723059±392219 | 10180±1415 | 179129±2905 | 1695833±356867 | 212100±1696 |
|  |  | 50 | 16708873±1369159 | 821330±191714 | 423190±127268 | 347839±27439 | 672914±8 | 29947±2864 |
|  | Drought stress | 0 | 19126783±538128 | 2255330±338352 | 52005±27523 | 512266±49924 | 976411±103041 | 15069±94 |
|  |  | 25 | 17116435±875282 | 746993±71275 | 940773±128489 | 321095±409567 | 773729±47759 | 24433±5604 |
|  |  | 50 | 16451150±3443174 | 1302479±196061 | 15111±6021 | 493501±127957 | 1409769±88878 | 44470±9190 |
| *T. serpyllum* | Regular watering | 0 | 14477094±739314 | 43863±6732 | 936677±172357 | 570769±168913 | 2049043±55584 | 21617±1078 |
|  |  | 25 | 13066912±203435 | 118716±29483 | 1196074±270681 | 242060±21768 | 3180274±138383 | 6064±20 |
|  |  | 50 | 14330038±846509 | 87881±828 | 159075±47395 | 89636±34358 | 3930232±168011 | 3678±2787 |
|  | Drought stress | 0 | 14364024±634644 | 1181069±246815 | 2637753±47539 | 459216±5034 | 1646945±123337 | 18198±253 |
|  |  | 25 | 13697332±499553 | 740981±188115 | 1235397±224319 | 678488±124425 | 1141713±19188 | 14390±1060 |
|  |  | 50 | 14370187±514043 | 224711±56250 | 822074±192139 | 380961±34618 | 2255163±300494 | 8426±970 |
| *T. kotschyanus* | Regular watering | 0 | 10532871±1645421 | 74108±9869 | 42616±21687 | 57325±16431 | 1370709±280659 | 14092±1732 |
|  |  | 25 | 11896739±838701 | 614971±150609 | 27042±8184 | 50042±57986 | 1736541±5053 | 11618±2531 |
|  |  | 50 | 9643620±1337828 | 1635652±16884 | 106066±11169 | 26987±1638 | 1702229±16650 | 4701±245 |
|  | Drought stress | 0 | 13987243±1213650 | 480067±484 | 1262709±136426 | 355625±40678 | 2018735±185727 | 26655±1346 |
|  |  | 25 | 13067441±1095645 | 1105031±258877 | 708926±265438 | 319933±51723 | 1198022±132874 | 14729±820 |
|  |  | 50 | 13606189±1571894 | 1149251±79955 | 1820448±57119 | 251282±22098 | 1302583±14421 | 15906±989 |
| LSD 5% |  |  | 1397825 | 167670.5 | 128607 | 65665.7 | 164376.7 | 2844.07 |

∗nd: non detected

**Supplementary Table 5C.** Phenolics content (peak area/mg FW) in thyme species after application of different concentrations of ABA and drought treatment.

| Species | Irrigation regimes(%fc) | ABA (µM) | Apigenin-glucuronide | Luteolin-glucuronide | Quercetin | 555-359 | 307-263 | 399-161 |
| --- | --- | --- | --- | --- | --- | --- | --- | --- |
| *T. vulgaris* | Regular watering | 0 | 3240477±586978 | 3236203±669150 | 22±3 | 3215606±315471 | 12058±2703 | 355624±30414 |
|  |  | 25 | 3482074±67413 | 2897112±8262 | 39±11 | 3408679±839890 | 14568±1583 | 1140952±19649 |
|  |  | 50 | 3429602±132856 | 2531422±84678 | 217±25 | 3699683±678522 | 17896±849 | 1611150±338595 |
|  | Drought stress | 0 | 4259273±291562 | 3617180±202094 | 214±12 | 3961643±424184 | 7600±1118 | 931810±70769 |
|  |  | 25 | 3266041±264742 | 2564331±128287 | 1130±78 | 3248267±956128 | 9919±2196 | 1132801±332126 |
|  |  | 50 | 4648603±2314 | 4043544±658610 | 73±10 | 2535232±522047 | 13206±886 | 1020097±228018 |
| *T. serpyllum* | Regular watering | 0 | 446824±38111 | 1007618±6620 | 11±2 | 640598±124437 | 14711±695 | 436792±84095 |
|  |  | 25 | 616558±55675 | 1271050±136189 | nd* | 390170±52047 | 9179±501 | 403713±55031 |
|  |  | 50 | 527499±94846 | 1592134± 281767 | nd | 274423±143382 | 5425±116 | 155511±22973 |
|  | Drought stress | 0 | 728068±21798 | 2052106± 253959 | 48±8 | 1971662± 444650 | 15528±2607 | 186820±25271 |
|  |  | 25 | 657536±93444 | 1121821±195647 | nd | 300546±54810 | 4262±382 | 233366±27984 |
|  |  | 50 | 449585±28494 | 772834±9677 | nd | 667174±2326 | 7611±151 | 334561±60650 |
| *T.*  *kotschyanus* | Regular watering | 0 | 432816±42630 | 1236316±172451 | 69±7 | 288703±38065 | 17178±504 | 167283± 8751 |
|  |  | 25 | 592421±61078 | 731855±3315 | nd | 191151±2810 | 13562±620 | 278156± 801 |
|  |  | 50 | 389988±2960 | 902788±163867 | nd | 54010±15958 | 6250±1097 | 86425±8565 |
|  | Drought stress | 0 | 882226±150026 | 1976543±293870 | nd | 621351± 15730 | 20032±993 | 133598±11978 |
|  |  | 25 | 665625±142959 | 1544023±344240 | nd | 231503±101319 | 19800±4496 | 122960±19064 |
|  |  | 50 | 432359±123861 | 1068807±98741 | nd | 250941±108012 | 12621±2550 | 142043±21497 |
| LSD 5% |  |  | 177096.9 | 270191.3 | 19.19 | 386067.4 | 1679.947 | 124295.8 |

∗nd: non detected

**Supplementary Table 6**. Concentrations of phytohormones (ng/g FW) in thyme species after application of different concentrations of ABA and drought treatment.

| Species | Irrigation regimes(%fc) | ABA (µM) | SA | SA-Gluc | Cis-OPDA | JA | JA-Ile | OH-JA-Ile | OH-JA | COOH-JA-Ile | Sulfo-JA |
| --- | --- | --- | --- | --- | --- | --- | --- | --- | --- | --- | --- |
| *T. vulgaris* | Regular watering | 0 | 99.13±6.71 | 172727.97±57523.74 | 871.76 ±108.07 | 1603.77± 111.29 | 21.92±5.86 | 5.08 ±0.46 | 247032.46±39617.56 | 6.38 ±1.78 | 15287849.10 ±1363367.79 |
|  |  | 25 | 79.87± 23.05 | 22545.02±4678.57 | 1088.91 ±175.70 | 1389.31±232.94 | 9.11 ±0.68 | 0.89 ±0.37 | 168570.75±11762.16 | 0.75 ±0.18 | 13905008.20±1182048.31 |
|  |  | 50 | 26.06 ±5.74 | 254641.32±25696.46 | 2067.02 ±9.18 | 528.48 ±18.39 | 3.30 ±0.68 | 0.64 ±0.18 | 211728.51±59652.57 | 1.23 ±0.10 | 16854846.20±1212859.10 |
|  | Drought stress | 0 | 36.26 ±6.68 | 89669.12±11019.46 | 2592.60 ±202.23 | 393.17 ±196.39 | 2.61 ±0.42 | 2.96 ±1.09 | 201159.37±35012.94 | 6.47 ±1.92 | 21339275.20±1371526.24 |
|  |  | 25 | 20.56 ±3.61 | 130827.58±20031.52 | 1452.08 ±70.29 | 441.18 ±221.48 | 1.47 ±0.38 | 0.92 ±0.32 | 163572.76±30050.56 | 1.36 ±0.45 | 14804055.40±216425.62 |
|  |  | 50 | 31.61 ±28.42 | 104101.12±30634.33 | 2983.30 ±839.59 | 803.74 ±334.77 | 1.75 ±1.18 | 1.41 ±0.22 | 101020.80± 19495.02 | 1.37 ±0.30 | 15923880.40±726539.92 |
| *T.serpyllum* | Regular watering | 0 | 122.86 ±16.77 | 416645.72±30540.53 | 2387.13± 374.99 | 41.56 ±21.67 | 5.65 ±1.57 | 2.10 ±0.28 | 35548.02±25545.82 | nd* | 15098.10 ±3230.37 |
|  |  | 25 | 186.94 ±40.61 | 349712.25±10672.58 | 1791.53 ±110.55 | 235.94 ±30.84 | 3.89 ±1.11 | 0.68 ±0.01 | 38066.24 ±2819.23 | nd | 13275.60 ±984.06 |
|  |  | 50 | 258.63 ±62.38 | 198175.38±77345.01 | 1128.58 ±6.37 | 53.26 ±8.10 | 3.57 ±0.45 | 0.71 ±0.28 | 39607 ±8207.65 | nd | 11121.20 ±1735.56 |
|  | Drought stress | 0 | 218.82± 117.08 | 687591.32±35483.66 | 2034.82 ±318.60 | 62.46 ±22.26 | 10.61±0.26 | 1.41 ±0.34 | 231286.61±42183.58 | nd | 7536.70 ±1736.84 |
|  |  | 25 | 418.01 ±36 | 531073.93±29258.91 | 1558.15 ±170.78 | 99.24 ±14.57 | 6.58 ±1.89 | 2.37 ±0.60 | 97509.50 ±1838.85 | nd | 9420.20 ±499.33 |
|  |  | 50 | 210.38± 68.61 | 273335.72±2275.81 | 2211.97 ±85.33 | 43.24 ±5.31 | 6.42 ±2.01 | 0.97 ±0.34 | 23970.43± 2901.68 | nd | 8233 ±645.24 |
| *T.kotschyanus* | Regular watering | 0 | 290.68 ±70.99 | 816335.81±175686.68 | 1475.20 ±366.80 | 1490.78±367.40 | 2.38 ±0.64 | 1.48 ±0.34 | 116583 ±12852.77 | 1.46 ±0.35 | 1256359.50± 23916.56 |
|  |  | 25 | 236.72± 59.72 | 192953.20±19934.40 | 1252.52 ±272.97 | 513.18 ±19.84 | 2.26 ±0.91 | 0.60 ±0.15 | 43787.09 ±863.43 | 0.62 ±0.02 | 2207692.50 ±14576.30 |
|  |  | 50 | 359.12 ±60.98 | 153645.24±18194.08 | 1859.12 ±7.64 | 1762.32±289.56 | 3.48 ±0.46 | 1.93 ±0.21 | 5280.07 ±2133.10 | 0.80 ±0.12 | 1511704.20 ±123466.37 |
|  | Drought stress | 0 | 831.35 ±154.60 | 315929.64±96239.06 | 2334.42 ±89.53 | 781.32 ±80.31 | 68.76 ±7.13 | 2.60 ±0.95 | 99763.26 ±17598.08 | 0.91 ±0.03 | 3770865 ±595551.18 |
|  |  | 25 | 415.09 ±39.27 | 240682.73±27294.32 | 805.09 ±3.16 | 357.71 ±43.69 | 3.87 ±0.67 | 0.90 ±0.21 | 168496.32±1785.61 | 0.66 ±0.02 | 3998015.60±239638.93 |
|  |  | 50 | 1100.97 ±176.32 | 456892.59±118320.10 | 2274.75 ±503.91 | 622.87 ±153.18 | 17.82 ±1.33 | 7.49 ±1.42 | 112971.23 ±13225.97 | 1.57±0.33 | 3687657.70 ±513616.84 |
| LSD 5% |  |  | 70.25 | 60115.86 | 282.95 | 163.60 | 2.30 | 0.53 | 24084.81 | 0.61 | 636696.40 |

∗nd: non detected

**Supplementary Table 7.** The results of principal components analysis for phytohormones of the thyme species.

| PC3 | PC2 | PC1 | Principal component |
| --- | --- | --- | --- |
| 1.1878 | 2.24573 | 2.85744 | Eigenvalue |
| 13.198 | 24.953 | 31.749 | Percent of Variance |
| 69.900 | 56.702 | 31.749 | Cumulative Percentage |
|  | | | |
| PC3 | PC2 | PC1 | Principal component for phytohormones |
| -0.0600856 | 0.546833 | -0.259759 | SA |
| -0.114052 | 0.2001 | -0.324674 | SA-Gluc |
| 0.83087 | 0.133332 | -0.0401531 | Cis-OPDA |
| -0.481094 | 0.181015 | 0.270593 | JA |
| 0.0293201 | 0.48936 | -0.027112 | JA-Ile |
| 0.0566244 | 0.567478 | 0.130012 | OH-JA-Ile |
| -0.0870272 | 0.0961554 | 0.417063 | OH-JA |
| 0.0378477 | 0.189704 | 0.506552 | COOH-JA-Ile |
| 0.220257 | -0.0604307 | 0.551457 | Sulfo-JA |

## Supplementary Figures


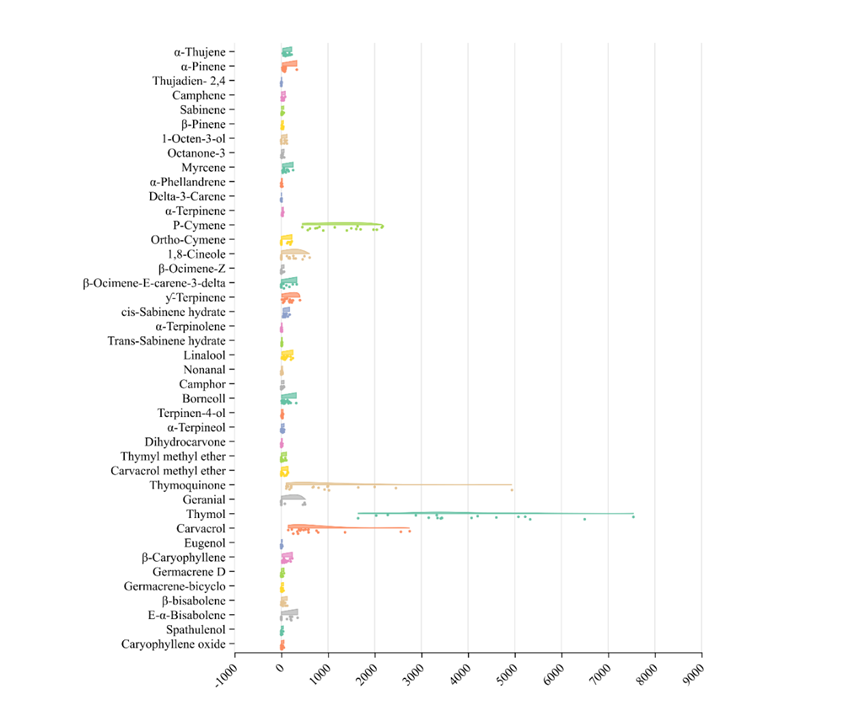


**Supplementary Figure 1.** Variation analysis of terpenoid components from three thyme species (*T. vulgaris*, *T. serpyllum* and *T. kotschyanus*). Terpenoids were measured by GC-MS (The values are expressed in ng/g of sample fresh mass) and annotated with NIST and ADAMS databases.
